# One‐Year Color Stability of a Reduced 37.5% Hydrogen Peroxide In‐Office Bleaching Protocol: A Randomized Clinical Trial

**DOI:** 10.1111/jerd.70173

**Published:** 2026-04-29

**Authors:** Mariana Evangelista Santos, Danielle Araújo Izídio Carvalho de Azevedo, Virgínia Feitosa Nogueira Rocha, Sônia Saeger Meireles

**Affiliations:** ^1^ Postgraduate Program in Dentistry Federal University of Paraíba João Pessoa PB Brazil; ^2^ Department of Restorative Dentistry Federal University of Paraíba João Pessoa PB Brazil

**Keywords:** hydrogen peroxide, randomized controlled trial, tooth bleaching

## Abstract

**Objective:**

This randomized clinical trial evaluated the one‐year color stability and patient satisfaction regarding dental esthetic improvement after 37.5% hydrogen peroxide (37.5HP) in‐office bleaching with a reduced application protocol.

**Materials and Methods:**

Forty participants with a shade mean C2 or darker were randomly allocated into two treatment groups (*n* = 20): two (37.5HP2) or three (37.5HP3) 8‐min applications per clinical session. Three bleaching sessions were performed at one‐week intervals. Tooth color was measured using a spectrophotometer at baseline, 1 week (1 W), 6 months (6 M), and 1 year (1 Y) post‐bleaching. Esthetic self‐perception was assessed using a 6‐point visual analogue scale. Statistical analysis was performed using the Shapiro–Wilk, Student's *t*‐test, Tukey, Mann–Whitney, and Friedman tests (*p* < 0.05).

**Results:**

No significant differences were observed between 37.5HP2 and 37.5HP3 for Δ$E_{\mathrm{ab}}^{*}$, Δ*E*_00_, or ∆WI_D_ across the evaluation periods (*p* > 0.05). Although both treatment protocols remained effective at 1Y post‐bleaching, a significant color rebound was observed from the 6 M assessment (*p* < 0.05). Patient satisfaction with dental esthetics was comparable between groups (*p* = 0.3).

**Conclusions:**

Two and three 37.5% HP applications provided similar bleaching stability after one year. Patients remained highly satisfied with their smile, regardless of the number of gel applications.

**Clinical Significance:**

Clinicians may consider using a neutral 37.5% HP in‐office bleaching gel with two 8‐min applications per session, as it provides the same one‐year bleaching longevity as the manufacturer's recommended protocol of three applications.

## 1 Introduction

The demand for esthetic dental procedures is motivated by aspects beyond vanity, including the potential to improve individual self‐perception [1, 2]. Tooth bleaching can be considered a health‐promoting procedure, since health is defined as a state of complete physical, mental, and social well‐being, not merely the absence of disease or infirmity [3]. One of the major causes of anxiety may be related to personal appearance and, consequently, to the perception of tooth discoloration, which can lead to an unattractive self‐image [1, 4, 5]. However, social standards and individuals' high expectations strongly influence the perception of dental treatments outcomes [6, 7].

Tooth bleaching is an esthetic treatment commonly indicated for managing discolored teeth [8, 9, 10] and it has been associated with a positive impact on domains of oral health related to quality of life (OHRQoL), including psychological comfort and social disability [1, 11, 12, 13, 14, 15, 16, 17]. The measurement of this impact varies from non‐significant to moderate, demonstrating that bleaching treatment increases concerns about dental appearance [1, 4].

Several tooth bleaching agents are available on the market, including products exclusively for in‐office use by professionals and those suitable for at‐home application, with or without professional supervision [9, 18, 19, 20, 21, 22, 23, 24, 25, 26]. Bleaching agents vary in hydrogen peroxide (HP) or carbamide peroxide (CP) concentration, composition, pH, and application technique [6, 27, 28]. The growing patient demand for faster whitening results has increased interest in in‐office bleaching procedures. This technique is usually performed over two or three clinical sessions using highly concentrated HP agents (20%–40%) [10, 20, 29, 30, 31, 32]. The duration of each clinical session depends on the number of bleaching gel replenishments and the total contact time between the gel and the tooth surface [10, 33, 34].

In‐office bleaching procedures are often associated with a higher risk of adverse effects, mainly due to the use of highly concentrated oxidizing agents [20, 29, 30]. Studies have suggested that the pH of bleaching agents may influence these undesirable effects and recommend the use of gels with a stable neutral to alkaline pH [8, 35, 36, 37]. These bleaching gels are associated with a lower risk and intensity of tooth sensitivity, as well as lower alterations in dental structure compared with acidic gels used in in‐office or combined bleaching protocols [37, 38, 39]. Moreover, neutral gels promote less diffusion of oxygen free radicals toward the pulp than acidic gels, which may explain the lower incidence of post‐bleaching tooth sensitivity observed with these agents [8, 40, 41]. The use of bleaching gels with a neutral to alkaline pH also enables the modification of clinical protocols, allowing a reduction in the number of applications without compromising the esthetic outcome [20, 36].

Clinical trials comparing protocols with reduced application times to those recommended by the manufacturer have demonstrated equivalent whitening efficacy [20, 35, 36, 42]. However, none of these studies evaluated the longevity of the whitening effect achieved with the reduced‐time protocols. Therefore, this study aimed to assess the one‐year color stability and patient self‐perception after 37.5% hydrogen peroxide in‐office bleaching using two or three applications per session. The hypotheses tested in this study were that the reduced protocol for 37.5% hydrogen peroxide in‐office bleaching affects (1) the tooth color stability and (2) patient self‐perception compared with the usual protocol 1 year after treatment.

## 2 Materials and Methods

### 2.1 Ethics Approval and Study Design

This clinical trial represents the one‐year follow‐up of a previous study [20] that was approved (CAEE: 67437217.9.0000.5188) by the Ethics and Research Committee of the Federal University of Paraiba (UFPB) and registered in the Brazilian Clinical Trials Registry (REBEC) under registration number RBR‐6nv76g. All participants who met the eligibility criteria signed an informed consent form prior to their enrollment.

This two‐arm, parallel, randomized, double‐blind clinical trial (patients and tooth color evaluator) was conducted at the clinics of the School of Dentistry from the UFPB, João Pessoa, PB, Brazil. The study design followed the guidelines published by Consolidated Standards of Reporting Trials [43] and respected the principles of the Declaration of Helsinki. This study also followed the International Committee of Medical Journal Editors (ICMJE) clinical trials registration guidelines [44]. Since the methodology of this study was previously described [20], this article describes a summary of the methods.

### 2.2 Recruitment and Eligibility Criteria

Each participant completed a medical history form, and a dental prophylaxis was performed to remove extrinsic stains. Participants included in this study were at least 18 years old and all of them had good general and oral health. They were recruited through advertisements displayed in the university corridors, on the university website, and via the researchers' social media accounts. For follow‐up evaluations (1 week, 6 months, and 1 year), patients were contacted through phone calls and social network groups (WhatsApp, Facebook Inc., and Instagram).

**FIGURE 1:**
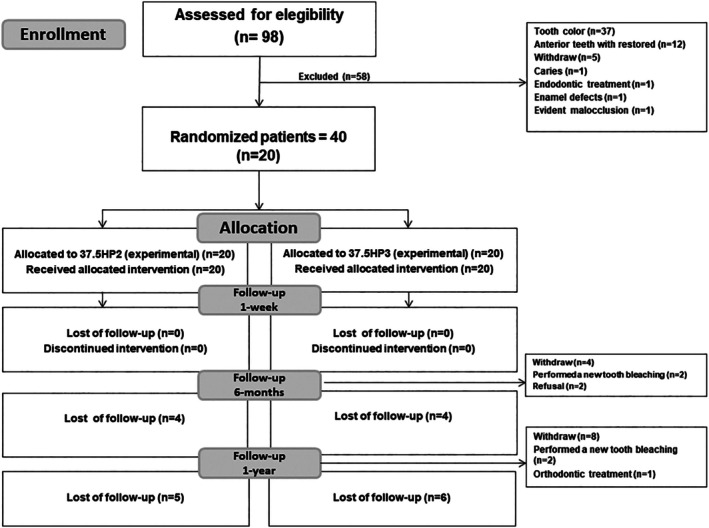
Flow diagram of the clinical trial.

To be included in the study, all six maxillary anterior teeth had to be present, with a shade mean of C2 or darker based on the value‐oriented shade guide (Vitapan Classical, Vita Zahnfabrik, Bad Säckingen, Germany) classification that was provided by the Vita Easyshade Advance spectrophotometer (Vita Zahnfabrik, Bad Säckingen, Germany). In addition, the anterior maxillary and mandibular teeth should not have more than 1/6 of the buccal surface covered with a restorative material. Exclusion criteria included patients presenting with or requiring treatment for active caries or periodontal disease, visible cracks in maxillary or mandibular anterior teeth, evident malocclusion, parafunctional habits, severe intrinsic tooth discoloration (such as tetracycline staining, fluorosis, or non‐vital teeth), tooth sensitivity, a history of previous bleaching treatment, or ongoing orthodontic therapy. Smokers, as well as pregnant or lactating women, were also excluded from the study (Figure 1).

### 2.3 Sample Size Calculation

The primary outcome of this study was the color change (Δ$E_{\mathrm{ab}}^{*}$), based on a previous study [42]. To detect a difference between groups of 2 units in Δ$E_{\mathrm{ab}}^{*}$, with 80% power, a 5% alpha error and, using a one‐tailed test, a minimum sample size of 16 participants/treatment group was required. To account for potential dropouts or refusals to participate, an additional 20% was added, resulting in a total sample size of 40 participants (20 in each treatment group).

### 2.4 Blinding, Randomization, and Allocation Concealment

Both the patients and tooth color evaluator were blinded to group allocation. Patients who met the inclusion criteria and agreed to participate were randomly assigned to two groups (*n* = 20) according to the number of 37.5% hydrogen peroxide (HP) applications: two (37.5HP2) or three (37.5HP3) 8‐min applications per clinical session (Pola‐Office Plus, SDI, Bayswater, Victoria, Australia). Simple randomization was designed and implemented by the research coordinator, who was not directly involved in the study interventions. A table was manually created to record participants' codes. Then, 20 cards labeled group 37.5HP2 and 20 labeled group 37.5HP3 were placed in a box, and the treatment allocation was randomly drawn and recorded for each participant code listed in the table. The allocation sequence was concealed in opaque, sealed envelopes numbered from 1 to 40, which were opened by the operator only at the beginning of each intervention.

### 2.5 Study Intervention

Before starting the treatment, participants received glasses for eye protection and underwent dental prophylaxis. The lips and tongue were isolated using a lip retractor (Arcflex FGM Dental Products, Joinville, SC, Brazil), and a light‐cured resin‐based gingival barrier was applied to protect the soft tissues (SDI, Bayswater, Victoria, Australia). A 37.5% HP gel with a pH of 7.4, evaluated by a pH meter with a micro pH electrode in the primary study of this research [20] was applied by the operator to the buccal surfaces of the incisors, canines, and premolars in both arches.

Participants in the 37.5HP2 and 37.5HP3 groups received two and three applications of the bleaching gel per clinical session, respectively. The whitening gel was applied according to the manufacturer's instructions and administered by the same operator, who was calibrated for all treatments. The product consists of a self‐mixing tube with a dispensing tip that facilitates accurate dosage and application of the gel. Each application lasted 8 min and was performed without the use of a light source. The bleaching gel was removed using high‐volume suction, and the teeth were rinsed with water between applications. A total of three clinical sessions were performed at one‐week intervals. All patients were instructed to brush their teeth regularly using a fluoridated toothpaste free of desensitizing or whitening agents.

### 2.6 Color Evaluation

After completing the anamnesis and clinical examination, a previously calibrated evaluator, who was not directly involved in the bleaching procedures and was blinded to the treatment groups, recorded tooth color at baseline using a spectrophotometer (VITA Easyshade Advance 4.0, Vita Zahnfabrik, BadSackingen, Germany) [45]. Prophylaxis was performed using a rubber cup with pumice and water before each tooth color evaluation. This procedure was repeated at 1 week, 6 months, and 1 year post‐bleaching. The color of the six maxillary anterior teeth was measured three times, with the active tip of the instrument positioned at the middle third of the buccal surface of each tooth. The device was calibrated before each evaluation. All measurements were performed freehand by an evaluator with 6 years of experience in tooth color assessment.

The spectrophotometer automatically calculated the mean of the three readings for each tooth. All assessments were carried out under standardized lighting conditions, in the same clinic at School of Dentistry, and at similar times of day (9:00–12:00). The device provides tooth shade measurements in two ways: according to the CIEL*a*b* color system or based on the 16‐shade tabs of a standard value‐oriented shade guide, arranged from 1 (highest value‐B1) to 16 (lowest value‐C4). The latter method was used for the randomization of participants into treatment groups.

Tooth color was determined according to CIEL*a*b* coordinates, where L* represents the value (lightness or darkness); a* is a measure of redness (positive a*) or greenness (negative a*); b* is a measure of yellowness (positive b*) or blueness (negative b*) [46]. The mean L*, a*, and b* values for each participant were obtained from the six maxillary anterior teeth. ΔL*, Δa*, and Δb* were calculated as the difference between post‐bleaching evaluation periods (1 week, 6 months, and 1 year) and baseline. Color differences between baseline and pos‐bleaching assessments were calculated using both CIEL*a*b* (Δ$E_{\mathrm{ab}}^{*}$) [33] and CIEDE2000 (Δ*E*_00_) formulas [47, 48, 49]. Additionally, color change was calculated as a function of the whitening index for dentistry (WI_D_), which is based on CIEL*a*b* coordinates and uses the following formula [50].

$${\mathrm{WI}}_{D}=0.511L^{*}-2.324a^{*}-1.100b^{*}$$

Higher WI_D_ values indicate whiter samples (whiteness), while lower WI_D_ values indicate darker samples (darkness). In addition, WI_D_ variations (∆WI_D_) can be assessed through comparison with 50:50% perceptibility and 50:50% acceptability thresholds for whiteness (WPT = 0.7; and WAT = 2.6, respectively) [48, 50, 51, 52, 53].

### 2.7 Assessment of Patient Satisfaction

Patients were asked to rate their satisfaction with improvements in dental esthetics at 1 week and 1 year post‐bleaching. A 6‐point visual analogue scale (VAS), ranging from 0 (no improvement) to 6 (exceptional improvement), was provided. Participants were instructed to select the single number that best represented their perception of the esthetic appearance of their teeth.

### 2.8 Statistical Analysis

The statistical analysis was conducted following the intention‐to‐treat principle and included all randomly allocated patients. Data were tested for normality using the Shapiro–Wilk test. Means and standard deviations of color change (Δ$E_{\mathrm{ab}}^{*}$, ∆E_00_, and ∆WI_D_) between baseline and the post‐bleaching assessment time points (1 week, 6 months, and 1 year) were normally distributed. Therefore, differences between groups were analyzed using Student's *t*‐test for independent samples. Median values and interquartile ranges of patient satisfaction in both treatment groups were compared using the Wilcoxon Signed Rank test. A significance level of 5% was adopted for all analysis.

## 3 Results

Ninety‐eight participants were initially examined, and 40 were included in this clinical trial and completed the treatment. Baseline characteristics of the participants were previously reported in the first publication related to this trial (Table 1) [20]. Eight participants missed the 6‐month follow‐up (four from each group). At the 1‐year recall, three additional participants were lost (one from the 37.5HP2 and two from the 37.5HP3 group). Reasons for missing the 6‐month and 1‐year recalls included relocation to another city (*n* = 6), undergoing a new tooth bleaching procedure before the 6‐month recall (*n* = 2), refusal to continue (*n* = 2), and initiation of orthodontic treatment (*n* = 1) (Figure 1).

**TABLE 1:** Descriptive characteristics of participants and baseline values of CIEL*a*b* coordinates according to treatment groups.

Characteristics		Treatment groups	
		37.5HP2	37.5HP3
Gender (*n*, %)	Male	6 (30)	8 (40)
	Female	14 (70)	12 (60)
Age in years (Mean, SD)		23 (2.9)	22.6 (3.0)
Education level in years of study (Mean, SD)		20 (1.7)	20.1 (1.6)
Profession (*n*, %)	Student	14 (70)	14 (70)
	Others	6 (30)	6 (30)
Color coordinate values (Mean, SD)	L*	82.1 (2.2)	82.4 (2.1)
	a*	0.2 (0.8)	0.2 (0.8)
	b*	23.8 (2.7)	23.0 (3.0)

*Note:* Table from the first published article of this study (Meireles et al. [10]).

Abbreviations: 37.5HP2, tooth bleaching performed with two applications/clinical session; 37.5HP3, tooth bleaching performed with three applications/clinical session; SD, standard deviation.

### 3.1 Color Evaluation

Results on bleaching efficacy for Δ$E_{\mathrm{ab}}^{*}$, ∆*E*_00_, and ∆WI_D_ at the 1‐week recall were previously reported [16]. At the 6‐month follow‐up, color rebound was observed in both treatment groups compared to the 1‐week post bleaching assessment (*p* < 0.01), with no significant differences between groups for Δ$E_{\mathrm{ab}}^{*}$, ∆*E*_00_, or ∆WI_D_ (*p* > 0.5). At the 1‐year recall, a slight increase in color rebound was noted for both groups in ∆*E*_00_ values (*p* < 0.05), and in the 37.5HP3 group for ∆WI_D_ (*p* < 0.003) compared to the 6‐month assessment. No significant differences were found between groups in Δ$E_{\mathrm{ab}}^{*}$, ∆*E*_00_, or ∆WI_D_ at the 1‐year recall (*p* > 0.05) (Table 2).

**TABLE 2:** Means (standard deviation) of color differences (Δ$E_{\mathrm{ab}}^{*}$ and Δ*E*_00_) and whiteness index for dentistry (∆WI_D_) between baseline and evaluation periods (1 week, 6 and 12 months) for different treatment groups.

Color evaluation	Assessment times	Treatment groups		*p* *
		37.5HP2	37.5HP3	
Δ$E_{\mathrm{ab}}^{*}$	1 week	10.2 (2.7)^a^	9.7 (2.7)^a^	0.6
	6 months	8.2 (2.6)^b^	7.7 (1.9)^b^	0.5
	1 year	7.5 (2.3)^b^	7.8 (1.9)^b^	0.7
Δ*E*_00_	1 week	6.1 (1.9)^a^	5.8 (1.5)^a^	0.6
	6 months	4.7 (1.5)^b^	4.6 (0.9)^b^	0.7
	1 year	4.2 (1.3)^c^	4.5 (0.9)^c^	0.5
ΔWI_D_	1 week	15.2 (5.7)^a^	14.1 (4.0)^a^	0.5
	6 months	12.6 (4.4)^b^	12.6 (3.1)^b^	1.0
	1 year	11.7 (4.3)^b^	11.9 (3.3)^c^	0.9

* Different lowercase letters indicate significant differences within the same treatment group in different assessment times (*p* < 0.05, paired student's *t*‐test).

### 3.2 Patient Satisfaction

At the 1‐year recall, the median (interquartile range) of patient satisfaction was 5 (5) in the 37.5HP2 group and 5 (4, 5) in the 37.5HP3 group. No significant differences were observed between groups (*p* = 0.3). However, a decrease in satisfaction with smile color was noted in patients from the 37.5HP3 group compared to the 1‐week post‐bleaching assessment (*p* = 0.03) (Table 3).

**TABLE 3:** Self‐perception scores of tooth appearance according to treatment groups and time‐intervals.

Assessment times	Treatment groups	
	37.5HP2	37.5HP3
1 week	6.0 (0.8)^Aa^	6.0 (0.8)^Aa^
6 months	6.0 (1.0)^Aa^	4.5 (1.4)^Ab^
1 year	6.0 (1.4)^Aa^	6.0 (1.2)^Ab^

*Note:* Median (standard deviation). Different capital letters indicate significant differences between treatment groups in the same evaluation period (*p* < 0.05, Mann–Whitney test). Different lowercase letters indicate significant differences within the treatment group at different evaluation periods (*p* < 0.05, Friedman test).

## 4 Discussion

Previous studies have suggested reducing either the application time or the number of gel replenishments during in‐office bleaching [20, 35, 36, 42] to decrease not only chair time and the amount of gel used, but also the risk of tooth sensitivity [21]. In the present study, we observed that reducing the number of a neutral 37.5% hydrogen peroxide applications per clinical session did not affect the one‐year color stability or patient self‐perception compared to the standard protocol recommended by the manufacturer. Consequently, both hypotheses tested in this study were rejected.

As in‐office bleaching is an effective procedure for treating tooth discoloration, the implementation of reduced protocols may also reduce treatment costs by decreasing the amount of bleaching gel used and chair time, potentially increasing accessibility to the population [18]. Previous studies have reported that shortening a single application from 40 to 30 min [35] or decreasing the number of 35% hydrogen peroxide gel replenishments from three to two [42] resulted in comparable bleaching efficacy while reducing the risk of tooth sensitivity. These findings support the growing trend toward shorter application times in in‐office bleaching protocols, which may improve patient comfort and satisfaction without compromising treatment outcomes.

The pH of bleaching agents is a factor frequently associated with tooth sensitivity [29, 35, 36, 38]. Neutral bleaching gels result in lower penetration of oxygen radicals into the pulp chamber compared to acidic gels [8], which may explain why these gels are associated with a reduced risk and intensity of tooth sensitivity, as well as a positive impact on treatment longevity [22, 39]. The pH stability of the bleaching gel can also influence the clinical protocol: unstable gels require multiple short applications, whereas neutral and stable gels can be applied in a single session with a longer exposure time or with fewer renewals, maintaining equivalent efficacy [23, 36, 38]. These benefits could be observed with the neutral whitening gel tested in this study [20], in both treatment protocols tested, with two or three applications/clinical session.

Although the penetration of acidic bleaching agents into tooth structures is greater than that of neutral or alkaline agents [8, 24], this does not appear to affect bleaching efficacy [37]. A clinical study reported no difference in color stability 1 year after treatment with 35% hydrogen peroxide at different pH values (acidic vs. neutral) [29]. Moreover, another clinical trial using the same bleaching gel tested in the present study, but with a protocol of three 12‐min applications per session over two sessions, reported that color stability was maintained for all evaluated parameters 1 year post‐treatment [25]. In contrast, our findings showed that both bleaching protocols resulted in a color rebound effect after 6 months, which persisted at the one‐year evaluation for ∆*E*_00_ (37.5PH2 and 37.5PH3 groups) and for ∆WI_D_ (37.5PH3 group). Consistent with these results, another clinical trial evaluating 40% HP in‐office bleaching (pH > 7.0) also reported color rebound 6 months after treatment, despite the higher agent concentration and longer exposure time [26].

One year after treatment, the whitening effect remained clinically relevant for both tested protocols. However, from the six‐month evaluation, a regression of the bleaching effect was observed, which continued up to 1 year, as reflected by reductions in Δ*E*_00_ and ΔWI_D_ in both groups. The reduced protocol exhibited a color rebound exceeding the perceptibility and acceptability thresholds reported in the literature for Δ*E*_ab_, Δ*E*_00_, and ΔWI_D_, whereas the traditional protocol showed color regression above the perceptibility thresholds [52, 53]. A previous study also reported color rebound 6 months after in‐office bleaching with 18%, 25%, or 40% hydrogen peroxide, with no significant differences among the tested concentrations [26]. In contrast, a long‐term clinical trial found that the efficacy of 35% hydrogen peroxide in‐office bleaching remained clinically acceptable 4.5 years after treatment, with a high overall color change from baseline and teeth considerably whiter than their initial condition [28]. Another clinical trial that used the same bleaching agent reported results similar to those found in our study [31]. The authors observed a slight rebound in tooth color 1 year after bleaching. However, the color rebound observed in our study was greater, which may be related to factors such as lighting conditions and the method used to standardize the spectrophotometer tip. In the previous study, a silicone matrix was used to standardize the location of the device tip, which may have interfered with the assessment by altering light transmission and background color during measurements [45]. In contrast, our study employed a freehand measurement approach performed by a professional with 6 years of experience using the device.

Tooth color rebound after bleaching is a predictable consequence of the natural aging process, in which gradual enamel loss increases dentin exposure and promotes progressive tooth yellowing. The combined effects of enamel thinning and secondary dentin formation may contribute to this rebound; however, the available evidence does not clearly establish its onset, rate of progression, or whether bleaching procedures intensify these age‐related changes. Moreover, tooth color stability is multifactorial and influenced by enamel wear, dentinal alterations, dietary patterns, and oral hygiene practices, making long‐term color predictability inherently limited [28].

Tooth bleaching has become a widely sought‐after procedure for improving dental esthetics in adults, with a significant impact on patients' esthetic perception, psychosocial well‐being, and overall quality of life [1, 6, 13]. After 1 year of treatment, patients reported remaining satisfied with the appearance of their teeth, regardless of the whitening protocol used. This finding highlights the positive impact of bleaching treatment on patients' quality of life, considering that other factors influencing smile esthetics—such as alignment, crowding, presence of fractures, tooth size, and previous restorations—were not individually evaluated [2, 14]. In this context, tooth bleaching should not be considered merely as a procedure associated with vanity, but rather as an intervention capable of contributing to the individual's general health [15, 25, 29].

In the present study, 65% of participants were young adult females aged 18–29 years. In general, this population tends to be more demanding regarding esthetic self‐perception compared with middle‐aged adults [16]. Previous studies have reported greater whitening efficacy among young adults [6, 16], which may be attributed to the higher organic content of dentin in younger individuals or to the greater presence of extrinsic pigments that can be more easily oxidized by bleaching agents [32]. These factors may help explain why, 1 year after treatment, teeth remained significantly lighter than at baseline, despite the color rebound observed 1 week post‐treatment. With advancing age, dentin thickness increases due to mineral deposition and the occlusion of dentinal tubules, reducing permeability to hydrogen peroxide and consequently diminishing bleaching effectiveness [17]. Moreover, each additional year of age has been associated with a 7% reduction in bleaching efficacy [32].

The lack of a subjective color assessment using a shade guide represents a limitation of this study. This limitation was primarily due to the restricted number of operators available to conduct the clinical procedures, which precluded the inclusion of a subjective color evaluation. Although digital spectrophotometry provides objective and highly reproducible measurements, visual shade matching remains widely used in clinical practice and reflects patients' and clinicians' real perception of color changes. Therefore, the lack of subjective evaluation may limit the direct clinical interpretation of our findings [20, 35, 50, 51]. Additionally, the exclusive use of instrumental assessment may have reduced variability associated with human perception; however, it also precludes direct comparisons with studies that incorporate both visual and instrumental methods [20, 35, 37]. Therefore, the results of the present study should be interpreted considering—and compared with—studies that employed objective color assessment methods, such as digital spectrophotometers.

Another limitation of this study was the use of only one bleaching gel formulation with a neutral pH. Although this choice allowed for better standardization of the protocol and control of confounding variables, it restricts the external validity of the results, as different formulations‐ such as acidic or alkaline bleaching gels‐ may present distinct efficacy and tooth sensitivity outcomes [37, 38, 41]. As previously discussed, pH can influence both the biological response and bleaching dynamics [24, 39, 40]. Nevertheless, the findings of this study should not be generalized to all bleaching agents. Two applications of 37.5% hydrogen peroxide per clinical session were sufficient to achieve high whitening efficacy and patient satisfaction 1 year after treatment. Thus, reducing the exposure time to bleaching agents may minimize the risk of tooth sensitivity, biological damage, and financial costs without compromising treatment effectiveness. However, further long‐term randomized controlled clinical trials are needed to evaluate in‐office bleaching protocols using different hydrogen peroxide concentrations and across diverse age groups.

## 5 Conclusion

Within the limitations of this study, 1 year after treatment, it can be concluded that:

1. The reduced protocol for 37.5% hydrogen peroxide in‐office bleaching demonstrated similar efficacy to the protocol recommended by the manufacturer. Although a gradual color rebound was observed in both protocols, the whitening effect remained clinically perceptible and satisfactory.
2. The reduced protocol for 37.5% hydrogen peroxide in‐office bleaching resulted in patient satisfaction comparable to that observable with the protocol recommended by the manufacturer.

## Funding

The authors have nothing to report.

## Disclosure

The authors have nothing to report.

## Ethics Statement

This clinical trial was approved (CAEE: 67437217.9.0000.5188) by the Ethics and Research Committee of the Federal University of Paraiba (UFPB) and registered in the Brazilian Clinical Trials Registry (REBEC) under registration number RBR‐6nv76g. All procedures performed in studies involving human participants were in accordance with the ethical standards of the institutional and/or national research committee and with the 1964 Helsinki Declaration and its later amendments or comparable ethical standards. The participants who meet the eligibility criteria signed an informed consent form before enrollment in the study. Details that might disclose the identity of the subjects under study were omitted.

## Consent

Informed consent was obtained from all individual participants included in the study.

## Conflicts of Interest

The authors declare no conflicts of interest.

## Data Availability

The data that support the findings of this study are available on request from the corresponding author. The data are not publicly available due to privacy or ethical restrictions.
